# Association between nonalcoholic fatty liver disease and subclinical atherosclerosis: a cross-sectional study on population over 40 years old

**DOI:** 10.1186/s12872-018-0877-2

**Published:** 2018-07-16

**Authors:** Jilin Zheng, Yong Zhou, Kuo Zhang, Yu Qi, Shimin An, Siyuan Wang, Xingquan Zhao, Yi-Da Tang

**Affiliations:** 1 Department of Internal Medicine, Coronary Heart Disease Center State, State Key Laboratory of Cardiovascular Disease, Fuwai Hospital, National Center for Cardiovascular Diseases, Chinese Academy of Medical Sciences and Peking Union Medical College, No. 167 Beilishi Road, Beijing, 100037 China; 2 Institute of Heart, Lung and Blood Vessel Diseases, Beijing Anzhen Hospital, Capital Medical University, Beijing, 100029 China; 3 Department of Neurology, Beijing Tiantan Hospital, Capital Medical University, Beijing, 100050 China

**Keywords:** Nonalcoholic fatty liver disease, Subclinical atherosclerosis, Carotid intima-media thickness, Brachial-ankle pulse wave velocity, Metabolic syndrome

## Abstract

**Background:**

Nonalcoholic fatty liver disease (NAFLD) refers to fatty infiltration of liver in the absence of excessive alcohol abuse. However, the problem that whether NAFLD is correlated with subclinical atherosclerosis assessed by carotid intima-media thickness (CIMT) and brachial-ankle pulse wave velocity (ba-PWV) remains a source of controversy. This can be attributed to the differences in diagnosis methods, population ethnicity, sampling size and bias. This study aimed to further investigate the association of NAFLD with subclinical atherosclerosis.

**Methods:**

A cross-sectional study was carried out in the current study on population aged over 40 years derived from Kailuan community-based prospective study among Chinese adults from June 2010 to June 2011. NAFLD was evaluated through ultrasonography and histories of alcohol consumption. Clinical parameters and medical histories of patients were collected in the manner of interview performed by trained investigators using the standardized questionnaires. The biochemical parameters were analyzed at the central laboratory. CIMT and ba-PWV of each patient were measured. Multivariate logistic regression was used to analyze the associations of NAFLD with subclinical atherosclerosis assessed by CIMT or ba-PWV.

**Results:**

A total of 4112 participants aged over 40 years were enrolled from Kailuan cohort, including 2229 men and 1883 women. The overall prevalence of NAFLD was 38.2% in the total population. Statistically significant differences were found in CIMT (*P* < 0.0001) and ba-PWV (*P* = 0.0007) according to the presence of NAFLD. It is notably that the multivariate logistic regression revealed NAFLD was independently associated with elevated CIMT after adjusting the conventional cardiovascular and metabolic risk factors (OR = 1.663, 95% CI = 1.391–1.989, P < 0.0001). In addition, NAFLD was also found to be positively associated with elevated ba-PWV after adjusting age, gender, BMI, current smoking and regular exercising (OR = 1.319, 95% CI = 1.072–1.624, *P* = 0.0089).

**Conclusions:**

Our findings suggest that NAFLD is remarkably correlated with subclinical atherosclerosis, which should be strongly advised to engage in the preventive strategies for cardiovascular diseases (CVDs).

## Background

Nonalcoholic fatty liver disease (NAFLD) is defined as fatty infiltration of liver in the absence of excessive alcohol abuse, which is suggested to be the most common cause of chronic liver disease worldwide. NAFLD is reported to affect up to 30% adults in western countries and 23.3% in the Chinese population [1–3].

Recent studies have focused on the relationship of NAFLD with cardiovascular diseases (CVDs) by means of their markers such as carotid intima-media thickness (CIMT) for arterial wall thickening [4, 5] and brachial-ankle pulse wave velocity (ba-PWV) for stiffening [6–8], as well as endothelial dysfunction [9], higher prevalence of vulnerable coronary plaques [10, 11], coronary artery calcification and abdominal aortic calcification [12]. However, the problem that whether NAFLD is associated with subclinical atherosclerosis assessed by CIMT and ba-PWV has not been clearly revealed, which remains controversial. These controversial results may be attributed to the differences in diagnosis methods, population ethnicity, sampling size and bias [13–16].

The current clinical gold standard for diagnosis of NAFLD is liver biopsy, however, it may be complicated by morbidity and even death [17], which is also not practical for screening the millions of individuals or for monitoring changes in fibrosis stage over time [18]. Magnetic resonance spectroscopy, which is the most precise method for quantification of triglyceride (fat) content in the liver [19], is time consuming to perform and restricted in spatial coverage requiring additional equipment and special expertise [20]. Considering ultrasonography is widely available, safe, well tolerated, relatively inexpensive and can be performed on scanners of any manufacturer, we chose it in this cross-sectional study to perform on a population aged 40 years or older for detecting fatty liver and CIMT, so as to further investigate the association of NAFLD with subclinical atherosclerosis.

## Methods

### Study design and populations

**Fig. 1:**
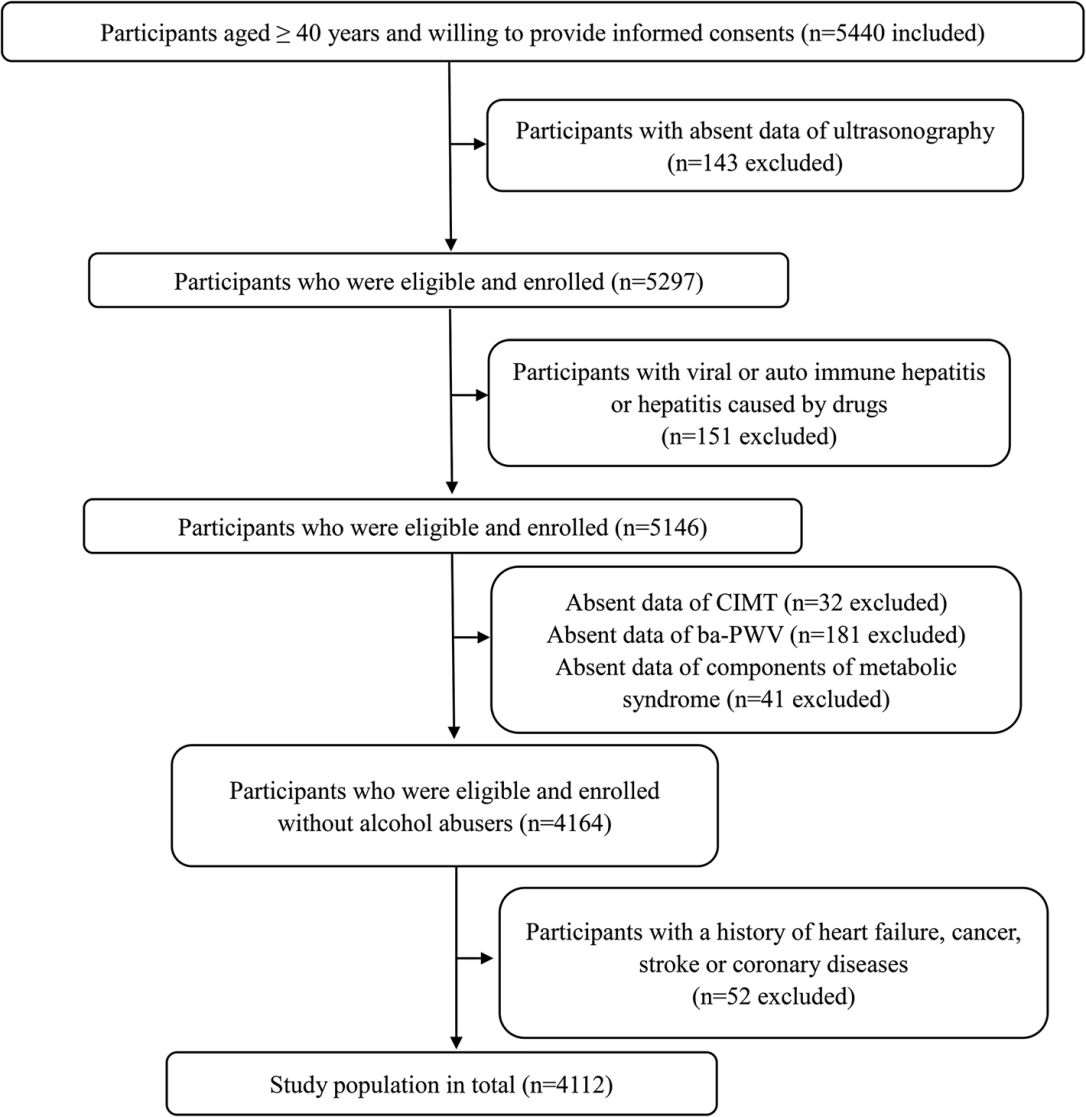
Flow chart of study population selection. Patients identified as potentially eligible who had provided the consent to participate and were included in the final analyses were shown in the figure. Ba-PWV, brachial-ankle pulse wave velocity; CIMT, carotid intima-media thickness; MetS, metabolic syndrome

As described previously [21], by means of a stratified random sampling approach by age and gender based on the data of the Chinese National Census from 2010, a sample of 7000 participants older than 40 years was randomly selected from the Kailuan cohort that included a total of 101,510 employees and retirees of the Kailuan (Group) Co. Ltd. between June 2010 and June 2011. The sample size was calculated based on detection of a 7% event rate with 0.7% precision and an α value of 0.05. The response rate was assumed to be > 80%. A total of 5440 people eventually agreed to participate in this study and provided the informed consents for baseline data collection. Among these 5440 participants, individuals with incomplete data, or viral or autoimmune hepatitis, or drug-induced hepatitis, or a history of cancer, heart failure, stroke or coronary diseases, as well as alcohol abusers were excluded. Finally, altogether 4112 participants were recruited into the current study. All data were processed using the Ruichi Precision Medical Record System, which was developed to standardize, integrate, manage, and analyze the precision medical data (Fig. 1).

### Clinical diagnosis of NAFLD

According to the Asia-Pacific Working Party on NAFLD and Chinese Association for the Study of Liver Disease [22, 23], NAFLD was diagnosed based on the presence of at least two of the following abnormal findings, namely, a) diffusely increased echogenicity of liver relative to kidney; b) ultrasound beam attenuation; and c) poor visualization of intrahepatic structures, after the exclusion of excessive alcohol abuse and other liver diseases. The severity of steatosis was differentiated by ultrasonography: slight (diffuse increase in fine echoes in liver parenchyma), moderate (diffuse increase in fine echoes with impaired visualization of the intrahepatic vessel borders and diaphragm), and severe (diffuse increase in fine echoes with non-visualization of the intrahepatic vessel borders and diaphragm). Abdominal ultrasonography was performed by experienced radiologists using a high-resolution B-mode topographic ultrasound system with a 3.5 MHz probe (ACUSON X300, Siemens, Germany). The radiologists were blinded to both clinical presentation and laboratory findings.

### Assessment of clinical and biochemical parameters

As was described in detail previously [21], the interview was performed by the trained investigators using a standardized questionnaire with questions on demographic and socioeconomic background, educational level, history of major diseases, alcohol consumption and smoking. Height was measured to the nearest 0.1 cm using a tape rule, and weight was measured to the nearest 0.1 kg using calibrated platform scales. BMI was calculated as body weight (kg) divided by the square of height (m^2^). Smoking was defined as smoking for at least one cigarette per day for over a year. Waist circumference (WC) was measured at umbilicus level at the late exhalation phase in standing position. Alcohol consumption was defined as at least 20 g/day for men and 10 g/day for women for over a year. Blood pressure was measured using a mercury sphygmomanometer. Readings of systolic blood pressure (SBP) and diastolic blood pressure (DBP) were taken twice at a five-minute interval, during which the participants had rested on a chair. The average of these two readings was used for current analyses. Notably, an additional reading would be taken in the presence of an over 5 mmHg discrepancy between these two measurements. The average of these three readings was used for further analyses. Arterial hypertension was defined as a systolic pressure of ≥140 mmHg or a diastolic pressure of ≥90 mmHg. Other biochemical variables were measured using an autoanalyzer (Olympus, AU400, Japan) at the central laboratory.

CIMT was measured by two experienced sonographers using a high-resolution B-mode tomographic ultrasound system (ACUSON X300, Siemens, Germany), with a linear 10-MHz transducer. Both sonographers were blinded to clinical presentation and laboratory findings of participants. Each participant had undergone bilateral carotid artery duplex sonography. Typically, the maximum CIMT was measured in the posterior walls of common carotid arteries on both sides 2 cm in front of the bifurcation, which was indicated by the distance between the first and the second echogenic lines of the anterior and posterior arterial walls. Particularly, images were focused on the posterior wall of common carotid artery, image quality was optimized using the gain setting, and the measurement was performed vertical to the arterial wall to accurately record the CIMT. The greater values of the right and left common CIMT were used for analysis.

After 10-15 min of rest, ba-PWV in all participants was measured by two experienced doctors using an arteriosclerosis detection device (Colin VP-1000, Model BP203RPE III; Omron, Tokyo, Japan). Both doctors were blinded to the clinical characteristics of participants. The pulse waves were measured simultaneously by placing cuffs on the right or left upper arm and the right or left ankle. Differences in the start times of pulse waves were corrected for distance. The greater values of right and left ba-PWV were used for further analyses.

According to the guidelines from the US National Cholesterol Education Program Adult Treatment Panel III [24], MS was defined as having ≥3 of the following metabolic risk factors, which were (1) central obesity: WC of ≥80 cm in women and ≥ 90 cm in men; (2) low high density lipoprotein cholesterol (HDL-C): fasting serum HDL-C of < 1.29 mmol/L in women and < 1.04 mmol/L in men; (3) high triglyceride (TG): fasting serum TG of ≥1.69 mmol/L; (4) high blood pressure (BP): BP of ≥130/85 mmHg or taking regular antihypertensive medications; and (5) high fasting blood glucose (FBG): FBG of ≥5.6 mmol/L or already taking anti-diabetic treatment. The recommended cut-points for WC had varied among different ethnic groups, which were modified to the Asian standard according to the Consensus Statement from the International Diabetes Federation [25].

### Statistical analyses

Data were processed using the SAS software (version 9.3; SAS Institute, Cary, North Carolina, USA). Categorical variables were compared using chi-square test, while continuous variables were analyzed by t-test. CIMT and ba-PWV were treated as dichotomous variables, with CIMT of 0.8 mm and ba-PWV of 1800 cm/s being treated as the cut-off values. The associations of NAFLD with CIMT or ba-PWV were analyzed using multivariate logistic regression in four different models by calculating the odds ratios (ORs) and 95% confidence interval (CI). Age, gender, BMI, regular exercising, current smoking status, WC, TG, LDL-C, diabetes mellitus and hypertension in different models were adjusted, since they might be the potential confounding factors in this study [15, 26–28]. Variance inflation factor was used to perform a multicollinearity diagnostic for the variables included in the models. All statistical tests were 2-tailed. Difference of *P* < 0.05 was deemed as statistically significant.

## Results

### Descriptive characteristics of participants

The overall prevalence of NAFLD in the finally included 4112 participants was 38.2%, which was 40.4% in men and 35.6% in women, respectively. The baseline characteristics and their distributions among participants with or without NAFLD were shown in Table 1. There were 999, 490 and 82 participants with a slight, moderate or severe steatosis, respectively. Elevated CIMT (≥0.8 mm) was found in 2498 (4.0%) participants, including 1095 (43.8%) in those with NAFLD and 1403 (56.2%) without NAFLD, respectively. Besides, abnormal ba-PWV (≥1800 cm/s) was found in 982 participants (23.9%), including 562 (57.2%) in those with NAFLD and 420 (42.8%) without NAFLD, respectively. Notably, both CIMT (P<0.0001) and ba-PWV (*P* = 0.0007) had displayed statistically significant differences in presence of NAFLD. In addition, statistically significant differences were also found between NAFLD and non-NAFLD groups in respect to gender, BMI, SBP, DBP, WC, TG, TC, HDL-C, LDL-C, uric acid, alanine aminotransferase (ALT), FBG, hypersensitive C-reactive protein (hsCRP), MS, hypertension and diabetes mellitus.

**Table 1:** General Characteristics of Participants with and without NAFLD

	Total (*n* = 4112)	Without NAFLD (*n* = 2541)	With NAFLD (*n* = 1571)	*P* Value
Age, y	55.8 ± 12.1	55.6 ± 12.7	56.2 ± 11.2	0.1155
Female, n (%)	1883(45.79)	1221(64.37)	671(35.63)	0.0018
BMI, kg/m^2^	24.87 ± 3.27	23.71 ± 2.79	26.75 ± 3.11	< 0.0001
Current smoking, n (%)	1050(25.54)	626(59.62)	424(40.38)	0.0927
Regular exercising, n (%)	1442(35.07)	864(59.92)	578(40.08)	0.1752
SBP, mmHg	130.90 ± 20.20	128.4 ± 20.65	134.9 ± 18.76	< 0.0001
DBP, mmHg	82.18 ± 10.91	80.52 ± 10.74	84.86 ± 10.66	< 0.0001
WC, cm	85.71 ± 9.66	82.40 ± 8.85	91.05 ± 8.45	< 0.0001
TG, mmol/L	1.60 ± 1.28	1.32 ± 0.95	2.04 ± 1.59	< 0.0001
TC, mmol/L	5.03 ± 0.99	4.94 ± 0.96	5.18 ± 1.03	< 0.0001
HDL-C, mmol/L	1.62 ± 0.46	1.67 ± 0.48	1.53 ± 0.39	< 0.0001
LDL-C, mmol/L	2.62 ± 0.74	2.57 ± 0.72	2.69 ± 0.78	< 0.0001
Uric acid, mmol/L	284.68 ± 87.31	270.9 ± 81.94	307.0 ± 91.05	< 0.0001
ALT, IU/L	17.80 ± 12.76	15.93 ± 11.22	20.83 ± 14.41	< 0.0001
Carotid plaque, n (%)	1612(39.20)	967(59.99)	645(40.01)	0.0555
Homocysteine, μmol/L	15.54 ± 9.50	15.40 ± 9.53	15.77 ± 9.44	0.2237
FBG, mmol/L	5.56 ± 1.51	5.39 ± 1.36	5.83 ± 1.70	< 0.0001
hsCRP, mg/L	2.16 ± 4.33	1.93 ± 4.56	2.53 ± 3.92	< 0.0001
Ba-PWV, n (%)				0.0007
<1800 cm/s	3130(76.12)	1979(63.23)	1151(36.77)	
≥ 1800 cm/s	982(23.88)	562(57.23)	420(42.77)	
CIMT, n (%)				< 0.0001
CIMT<0.8 mm	1614(39.25)	1138(70.51)	476(29.49)	
CIMT≥0.8 mm	2498(60.75)	1403(56.16)	1095(43.84)	
Metabolic syndrome, n (%)	1097(26.68)	358(32.63)	739(67.37)	< 0.0001
Hypertension	1919(46.67)	993(51.75)	926(48.25)	< 0.0001
Diabetes mellitus	489(11.89)	214(43.76)	275(56.24)	< 0.0001

Data are means ± SD or median (interquartile ranges) or number (percentage) of subjects. ALT, alanine aminotransferase; Ba-PWV, brachial ankle pulse wave velocity; BMI, body mass index; CIMT, carotid intimamedia thickness; DBP, diastolic blood pressure; FBG, fasting blood glucose; HDL-C, high-density lipoprotein cholesterol; hsCRP, hypersensitive C-reactive protein; LDL-C, low-density lipoprotein cholesterol; NAFLD, nonalcoholic fatty liver disease; SBP, systolic blood pressure; TG, triglycerides; TC, total cholesterol; WC, waist circumference. The chi-squared test was used for comparison of categorical variables and t-test was used for continuous variables. The *P* value <0.05 was regarded as statistically significant

NAFLD was significantly associated with elevated CIMT (OR = 1.866, 95% CI = 1.633–2.131, *P* < 0.0001) and ba-PWV (OR = 1.285, 95% CI =1.111–1.487, *P* = 0.0008), respectively, as was shown in crude (unadjusted) model in Table 2. In addition, the associations remained significant in model 1 (CIMT: OR = 2.050, 95% CI =1.761–2.387, *P* < 0.0001; ba-PWV: OR = 1.343, 95% CI = 1.117–1.615, P = 0.0008) controlling for the effect of age and gender, as well as in model 2 (CIMT: OR = 1.781, 95% CI =1.501–2.112, P < 0.0001; ba-PWV: OR = 1.319, 95% CI = 1.072–1.624, *P* = 0.0089) adjusting for age, gender, BMI, current smoking status and regular exercising. Based on model 2, possible confounding cardiometabolic risk factors were adjusted in model 3, which showed that NAFLD remained to be associated with the elevated CIMT (OR = 1.663, 95% CI = 1.391–1.989, P < 0.0001). However, the association between NAFLD and elevated ba-PWV was not found statistically significant (*P* = 0.5601). No significant multicollinearity of variables included in the models was found after multicollinearity diagnosing by variance inflation factor.

**Table 2:** Association between NAFLD and CIMT and Ba-PWV in Different Logistic Regression Models

	CIMT		Ba-PWV	
	OR(95%CI)	*P* Value	OR(95%CI)	*P* Value
Unadjusted	1.866 (1.633–2.131)	< 0.0001	1.285 (1.111–1.487)	0.0008
Model 1	2.050 (1.761–2.387)	< 0.0001	1.343 (1.117–1.615)	0.0017
Model 2	1.781 (1.501–2.112)	< 0.0001	1.319 (1.072–1.624)	0.0089
Model 3	1.663 (1.391–1.989)	< 0.0001	1.068 (0.855–1.335)	0.5601

Model 1: Adjusted for age and gender; Model 2: Further adjusted for BMI, regular exercise and current smoking based on model 1; Model 3: Further adjusted for WC, TG, LDL-C, diabetes mellitus and hypertension based on model 2. Ba-PWV, brachial ankle pulse wave velocity; CIMT, carotid intimamedia thickness; NAFLD, nonalcoholic fatty liver disease; OR, Odds ratio; CI: confidence interval

## Discussion

A total of 4112 participants have been enrolled in the current study, and it is a remarkable fact that NAFLD is associated with increased ba-PWV (ba-PWV: OR = 1.319, 95% CI = 1.072–1.624, *P* = 0.0089) after adjusting for age, gender, BMI and lifestyle. Besides, NAFLD was found independently associated with CIMT (OR = 1.663, 95% CI = 1.391–1.989, *P* < 0.0001) after controlling for the conventional cardiovascular and metabolic risk factors.

Our findings are consistent with those from several previous studies, which have supported the associations of NAFLD with CIMT [4, 13, 29–31]. Juha Koskinen et al. reported that NAFLD assessed by elevated liver enzymes has been found to relate with CIMT independent of age, sex, and alcohol intake among 1553 participants. In another study involving 1021 participants, fatty liver diagnosed by ultrasound suggests that NAFLD is evidently associated with the elevated CIMT both in men and women after adjusting for the conventional cardiovascular risk factors and MS components [13]. In addition, Mohammadi et al. also reported that NAFLD was associated with the increased CIMT and CVD risk factors with/without MS among 335 participants [29]. Besides, studies evaluating NAFLD by liver biopsy also demonstrate that NAFLD is an independent risk factor of CIMT [30, 31]. However, there are several studies reporting the inconsistent results [15, 32]. For instance, Petit et al. reported that NAFLD diagnosed by MRI was not related to the elevated CIMT in a study restricted to type 2 diabetes mellitus (T2DM) [15]. In another diabetic heart study involving 623 participants with high prevalence of T2DM, fatty liver diagnosed by computed tomography scan was reported to be less likely to be a direct mediator of CIMT, which might instead represent an epiphenomenon [32]. Such inconsistency might be attributed to the heterogeneities in the diagnosis methods of fatty liver and the selection of special study population. Therefore, whether the impact of visceral adipose tissue and insulin sensitivity or other related factors has been adequately evaluated in the participants may give rise to different conclusions.

The current study also revealed that, NAFLD is distinctly associated with ba-PWV, which is independent of the conventional cardiovascular risk factors but not of MS. As is reported in a study recruiting 220 participants, fatty liver diagnosed by ultrasound suggests that NAFLD is a major independent contributor to arterial stiffness defined as PWV [15]. Vlachopoulos et al. also demonstrated that NAFLD evaluated by liver biopsy was correlated with PWV and endothelial dysfunction [33]. MS is a potential confounding risk factor, which shares similar associations such as hypertriglyceridemia, obesity and diabetes with NAFLD. Additionally, evidences also show that NAFLD may actually be a hepatic manifestation of MS, since metabolic risk factors are common in NAFLD patients. Nearly 90.0 and 33.0% NAFLD subjects have at least one character and all characters of MS, respectively [34]. Consequently, this may be responsible for the reason why NAFLD is markedly associated with ba-PWV independent of conventional cardiovascular risk factors, but shows no statistical significance after adjusting for MS. Nevertheless, evidences with larger sample size should be added to present the association of NAFLD with arterial stiffness assessed by ba-PWV.

This study adds to the knowledge about NAFLD and subclinical atherosclerosis in the Kailuan community-based population of age 40 years or older. However, the cross-sectional design of this study has limited our ability to conclude a cause–effect relationship between NAFLD and subclinical atherosclerosis. Therefore, more prospective studies with larger sample size are in urgent need to confirm this relationship.

## Conclusions

The current cross-sectional study is conducted (4112 screening from a total of 5440 participants derived from Kailuan cohort) to explore the relationship of NAFLD with subclinical atherosclerosis in population aged over 40 years. It is noteworthy that NAFLD is associated with increased ba-PWV after adjusting for age, gender, BMI and lifestyle. More importantly, NAFLD was found independently associated with elevated CIMT after further controlling for the conventional cardiovascular and metabolic risk factors. Therefore, NAFLD intervention may be served as a potential therapeutic target to prevent the incidence of CVDs.

## Abbreviations

ALT: Alanine aminotransferase
Ba-PWV: Brachial ankle pulse wave velocity
BMI: Body mass index
CI: Confidence interval
CIMT: Carotid intimamedia thickness
CVD: Cardiovascular disease
DBP: Diastolic blood pressure
FBG: Fasting blood glucose
HDL-C: High-density lipoprotein cholesterol
hsCRP: Hypersensitive C-reactive protein
LDL-C: Low-density lipoprotein cholesterol
MS: Metabolic syndrome
NAFLD: Nonalcoholic fatty liver disease
OR: Odds ratio
SBP: Systolic blood pressure
TC: Total cholesterol
TG: Triglycerides
WC: Waist circumference
